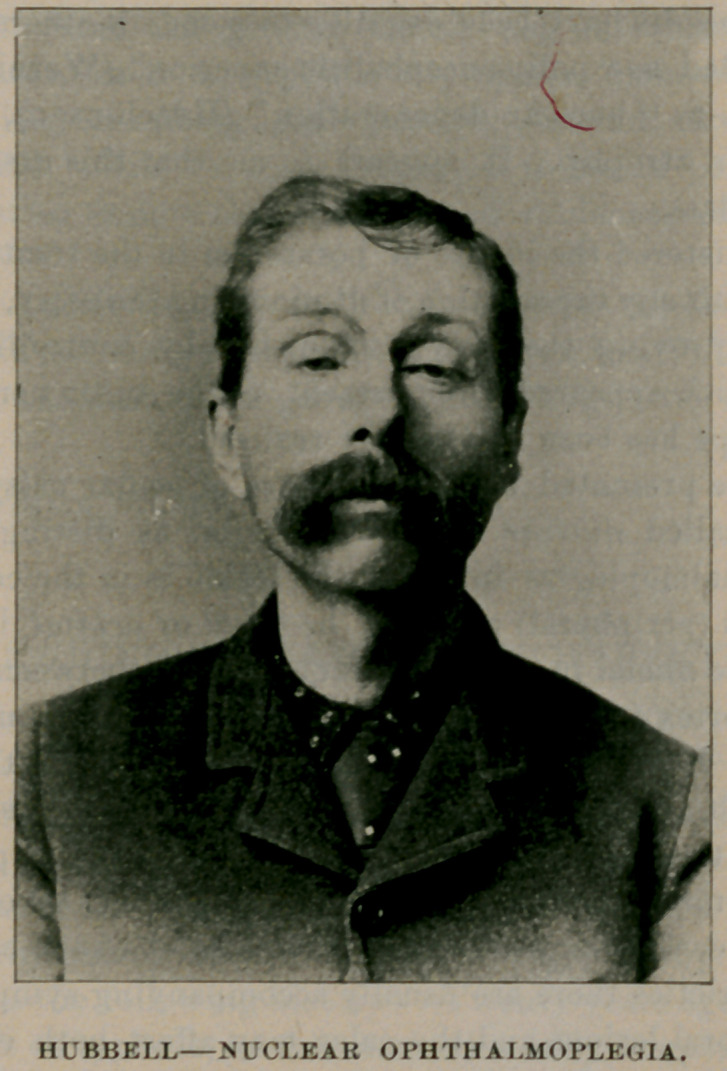# Nuclear Ophthalmoplegia

**Published:** 1895-08

**Authors:** Alvin A. Hubbell

**Affiliations:** Buffalo, N. Y., Professor of diseases of the eye and ear in the Medical Department of Niagara University; Surgeon to the Charity Eye, Ear and Throat Hospital; Oculist and Aurist to the Sisters of Charity Hospital, etc.


### NUCLEAR OPHTHALMOPLEGIA.

By ALVIN A. HUBBELL, M. D., Buffalo, N. Y.,

Professor of diseases of the eye and ear in the Medical Department of Niagara University; Surgeon to the Charity Eye, Ear and Throat Hospital; Oculist and Aurist to the Sisters of Charity Hospital, etc.

MR. H. W. offers a striking and typical picture of chronic, progressive or nuclear ophthalmoplegia. He is a farmer, now 40 years of age, in good health and with no history of syphilis or any severe illness. When he was twelve years old he had excessive hemorrhage from the nose. Soon afterward it was noticed that he could not turn his eyes as far as usual. This impairment of motion increased and for many years, he cannot tell how many, he has not been able to turn either eye in any direction. He consulted me first during the summer of 1892. He then presented the physiognomy shown in the accompanying engraving, which is from a photograph taken in 1893.[1] His eyeballs are directed horizontally forward and the visual axes are parallel. He is unable to turn his eyes to the slightest extent in any direction, from side to side, up or down, although he can converge the eyes slightly on looking at a

1. See Fig., p. 41.

nearby object. He cannot open his eyes except by the forcible action of the occipito-frontalis muscle and then only to lift the upper lids to the center of the cornea. He winks very infrequently, the winking reflex being very much impaired. The muscles of both sides of the face supplied by the facial nerve are stiff and inactive, although not completely paralysed. When he laughs, the facial expression is most unnatural, the ordinary movements being much restricted. Another peculiarity is that perspiration takes place about the face at ordinary temperatures while there is no perspiring of any other part of the body.

The pupils are somewhat larger than normal and respond very slightly to light, and not at all to efforts at convergence and accommodation.

As regards the state of vision it is somewhat below normal, that of the right eye being, when first tested in the summer of 1892, No. 24 Snellen, at 5 meters ($\frac{5}{24}$), and the left, No. 9 Snellen, at 5 meters ($\frac{5}{9}$). Under the influence of iodide of potassium for a year it improved somewhat, rising in the right eye to $\frac{5}{12}$ partly and the left to $\frac{5}{6}$ partly. There is slight myopia in both eyes, that of the right being 1.00 D., and the left 0.50 D. His power of accommodation seems to be preserved, as he is able to read ordinary and even fine print without difficulty at fourteen inches. The visual fields are normal and he seldom sees double.

Examination with the ophthalmoscope shows both fundi to be normal in appearance, excepting, perhaps, a slight obscuration of the outlines of the optic discs.

In brief, then, this case presents the following symptoms:

1. Immobility of both eyeballs, excepting,
2. Slight convergence power for near vision.
3. Ptosis, both eyes.
4. Pupils somewhat enlarged.
5. Pupil reflex nearly lost.
6. Action of ciliary muscle (accommodation) preserved.
7. Winking reflex much weakened.
8. Paresis of both facial nerves.
9. Hyperidrosis of the face.
10. Slight optic neuritis (?).

The course of the disease has evidently been progressive, the muscles of the eyeballs being affected first and afterward those of the face, which are not yet completely paralysed. All the external muscles of the eyes are paralysed almost completely, but those within the eye retain their function quite fully, as is evidenced by the power of the ciliary muscle to accommodate and the sphincter of the iris to preserve the nearly normal size of the pupil.

The etiology is obscure and although referred to the nose-bleed

I cannot conceive what relation the latter has to it. The history of the case entirely eliminates syphilis. As the patient is free from all symptoms pointing to other diseases, it is impossible to attribute it to a pathological condition to which it might be secondary.

The diagnosis in this case clearly locates the lesion in that group of nuclei lying beneath the aqueduct of Sylvius and in the floor of the fourth ventricle of the brain, from which the motor nerves of the eye and other cranial nerves take their origin. Be-

HUBBELL—NUCLEAR OPHTHALMOPLEGIA.

ginning near the posterior extremity of the third ventricle and running backward we have, first, the nuclei of the motor-oculi, or the third nerve, then that of the trochlear, patheticus, or fourth, next that of the abducens, or sixth, and lastly the facial, or seventh nerve. The most anterior nuclei are for the fibers of the third nerve which supply the iris and ciliary muscle and the muscles of convergence. The most posterior give origin to the facial together with those fibers supplying the orbicularis palpebrarum. In the case before us, therefore, the most anterior nuclei must have escaped, in a measure at least, the disease-process, as is shown by the nearly normal-sized pupils, the fair accommodation and the slight ability to converge for near vision. The most posterior nuclei were involved last, as the facial muscles have become affected more recently and still preserve their functions to a certain degree.

What this lesion is, which began a little behind the most anterior nuclei and has gradually extended backward, it is not easy to say. In cases where post-mortem examinations have been made, the results have been negative in some, in others they have been regarded as "polio-encephalitis superior" (Wernicke), and in others still as "nuclear degeneration" (Hutchinson), the same as in muscular atrophy. It appears to me that this case belongs to the latter class.

I administered the iodide of potassium in the treatment in this case, not with any expectation of diminishing the palsy, but with the hope of improving the vision somewhat by controlling a slight neuritis which appeared to be present in the optic nerves. I feel that my hope has been measurably realised.

We have presented to us here a case of ocular palsy of nuclear origin, so-called *nuclear* ophthalmoplegia, as distinguished from those ophthalmoplegias in which the lesion is in the course of the nerve-fibers (peripheral) and may be *basal* or *orbital* in situation, or above the nuclei in the intra-cerebral tract between the nuclei and the cortex (*fibrillar or cortico-peduncular*), or in the cortex (*cortical*). The characteristic features of nuclear ophthalmoplegia are set forth in the report of this case. The disease is progressive and affects the muscles of both eyes alike. In the peripheral forms one or few muscles may be involved and confined to one eye, and are usually easily diagnosticated by the history of the case. In the cerebral varieties there are usually accompanying symptoms pointing to cerebral lesions and the palsy may affect both eyes so as to produce conjugate deviations.

Cases of nuclear ophthalmoplegia are so infrequent and autopsies in such cases so rare that its study, etiologically and pathologically, is still very interesting. It has been found both as a primary affection and as secondary to other diseases, such as tabes, disseminated sclerosis, diphtheria, tuberculosis, diabetes, exophthalmic goitre, syphilis and poisons. It may also arise from traumatism of the head. Vascular lesions and disturbances of circulation may induce it. Dufor (*Annales d'Oculistique*, Mars-Avril, 1890, p. 97,) has made the most extensive collection of cases of nuclear palsies with which I am acquainted. His collection numbered 220. Out of these, 37 autopsies had been made, establishing that 9 were due to tumors, 8 to hemorrhage, 1 to softening following thrombosis, 7 to acute hemorrhagic inflammation, 3 to inflammatory degeneration, acute or chronic, 6 to atrophic degeneration and the others were negative. As to sex, out of 163 in which this was determined, 122 were males and 41 females. In 125, where the age was given, 23 cases were 1–15 years old; 35, 15–30 years, and 67, 30–69 years. Out of 183 cases the general health was enfeebled, in 105 by other diseases, such as syphilis, diphtheria, diabetes, locomotor ataxia, labio-glosso-pharyngeal paralysis, disseminated sclerosis, and the like, while 78 cases appeared to be in perfect health. It is interesting to note that Dufor found evidences of syphilis in 42 cases.

The prognosis depends upon the nature of the lesion and the cause and is not always hopeless. The treatment, therefore, may avail much and should be governed according to the etiology and diagnosis.

212 Franklin Street.

## Figures and Tables

**Figure f1:**